# A role for aquaporins in the modulation of cold stress tolerance in oriental melon

**DOI:** 10.1093/plphys/kiae578

**Published:** 2024-11-01

**Authors:** Maria-Angelica Sanclemente

**Affiliations:** Horticultural Sciences, University of Florida, Gainesville, FL 32601, USA; Assistant Feature Editor, Plant Physiology, American Society of Plant Biologists

Climate change significantly disrupts typical patterns of low temperatures worldwide, affecting the severity, duration, and frequency (Sippel et al. 2020). Long-term studies of climate model simulations indicated that extremely low temperatures are likely to persist throughout the 21st century, affecting regional and global cold seasons to different extents (Kodra et al. 2011). For instance, large fluctuations in low and high temperatures in the Midwest of the United States have impacted phenology and ecology of native forests, making infrequent frost damage events more common (Gu et al. 2008; Augspurger 2013). Most major crops in the world are grown in areas prone to freezing temperatures and frost injury. Understanding how plants cope with cold stress thus remains vital for plant cultivation and the overall health of ecosystems.

Cold stress temperatures range from chilling (0–15 °C) to freezing (<0 °C) (Bhat et al. 2022). These conditions normally alter growth and development as well as many cellular and biochemical processes (Ding et al. 2019). A prominent impact occurs at the cell membrane, where loss of integrity and fluidity triggers signaling pathways for downstream stress responses (Ding et al. 2019). Plants have evolved diverse mechanisms for low temperature sensing, including the Ca^+^-mediated Inducer of CBF Expression (ICE1)-C-repeat binding factor 3 (CBF)/Dehydration responsive element binding proteins (DREB1) path and upregulation of abscisic acid (ABA) and reactive oxygen species signals (Adhikari et al. 2022).

Soluble sugars have long been recognized to be essential for cold acclimation. Their accumulation contributes to membrane stability, osmo-protection, and regulation of metabolic signals. Recent studies showed that trehalose acts as a nexus between extracellular production of hydrogen peroxide (H_2_O_2_) and induction of antioxidant systems in the cytoplasm (Fig.; Liu et al. 2021). The trehalose–H_2_O_2_ signaling pathway enhances cold tolerance, yet the mechanistic understanding of the pathway is very limited. In this issue of *Plant Physiology*, Han et al. (2024) used a combination of biochemical probes, gene expression analysis, yeast 1-hybrid, and reporter assays to identify components of the trehalose-mediated cold tolerance pathway and their modes of actions.

**Figure.**
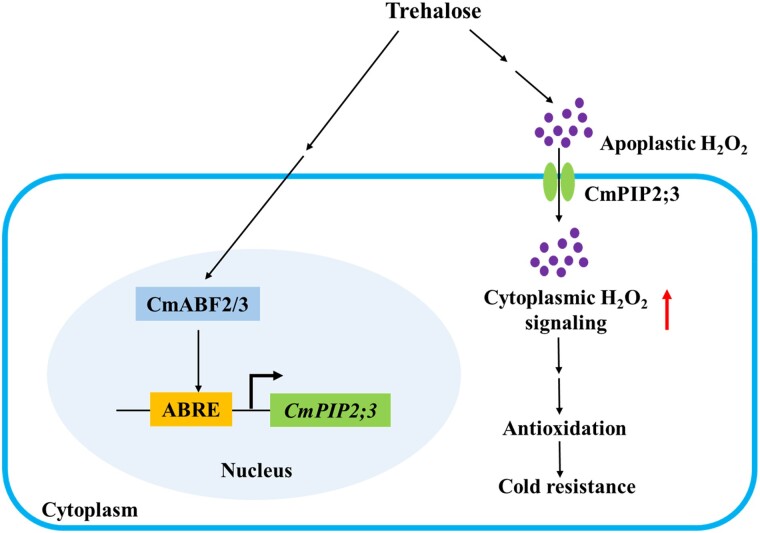
Contributions from trehalose and H_2_O_2_ to cold tolerance in oriental melon. A model for modes of action of trehalose, H_2_O_2_, *Cm*PIP2;3, and *Cm*ABF2/3 in the cold tolerance response of oriental melon seedlings. Figure was adapted from Han et al. (2024).

Initial analyses showed that trehalose treatment of cold-stressed melon seedlings [*Cucumis melo* (*Cm*) var. *makuwa* Makino] induced H_2_O_2_ accumulation in the apoplast. When H_2_O_2_ production was inhibited with diphenyleneiodonium, an inhibitor of NADPH oxidases, there was a significant increase of plant damage compared with trehalose- or H_2_O_2_-treated plants. Moreover, inhibition of H_2_O_2_ production significantly reduced the activities of ascorbate peroxidase and gluthatione reductase. The effects of diphenyleneiodonium were alleviated by either trehalose or H_2_O_2_ treatment, consistent with the suggested roles for endogenous H_2_O_2_ in modulating trehalose-induced cold tolerance.

Apoplastic H_2_O_2_ can be sensed by H_2_O_2_-induced Ca^2+^ (HPCA1) membrane receptor or can be transported into the cytosol through aquaporins (Foyer 2020). Han et al. (2024) showed that 2 plasma membrane–localized aquaporins, *Cm*PIP2;1 and *Cm*PIP2;3, modulate intracellular H_2_O_2_ transport and subsequent signal transduction to different extents.

Initial observations showed that expression of *CmPIP2;1* and *CmPIP2;3* is upregulated by exogeneous trehalose or low temperatures (15 °C/6 °C, day/night). Subsequent colorimetric assays with H_2_O_2_ specific probes Amplex Ultra Red or Amplex Red (AR), which have different membrane permeabilities, were used to detect transport capacity of *CmPIP2;1* and *CmPIP2;3*. For instance, H_2_O_2_-treated recombinant yeast cells expressing *CmPIP2;1* and *CmPIP2;3* showed red fluorescence emitions from the AR dye, indicating that these aquaporins could transport H_2_O_2_ from the media to the cells. In contrast, when *CmPIP2;1* and *CmPIP2;3* were silenced in melon seedlings (pTRV-*CmPIP2;1* and pTRV-*CmPIP2;3*) with the VIGS system, the ratio of the Amplex Ultra Red dye for extracellular H_2_O_2_ and the AR probe increased significantly compared with seedlings with the empty vector (pTRV). Consistently, cold-stressed pTRV-*CmPIP2;1* and pTRV-*CmPIP2;3* seedlings showed more leaf damage, high levels of electron leakage, increased malondialdehyde content, and reduced activities of ascorbate peroxidase and glutathione reductase. Furthermore, trehalose treatment failed to alleviate the observed responses, indicating that CmPIP2;1 and CmPIP2;3 alter trehalose-induced cold tolerance, H_2_O_2_ transport, and the downstream signals that mitigate stress through antioxidants activity (Han et al. 2024).

Previous studies have identified 5 trehalose-responsive abscisic acid stress–inducible bZIP transcription factors in melon (*Cm*ABF) associated with cold-tolerance (Liu et al. 2022). In Han et al. (2024) only the *CmPIP2;3* promoter was shown to contain ABA-responsive element motifs that allow binding of 2 *Cm*ABFs: *Cm*ABF2 and *Cm*ABF3. These 2 transcription factors regulated *CmPIP2;3* promoter, H_2_O_2_ transport, and trehalose responsiveness (Fig.). The contributions by *Cm*PIP2;1 and its associated mechanisms will require further exploration.

Many gaps remain in our understanding of cold stress in plants. The main path of H_2_O_2_ production is mediated by the NADPH oxidase respiratory burst oxidase homolog D (RBOHD). Trehalose upregulates *RBOHD* expression and H_2_O_2_ accumulation (Liu et al. 2022). However, it is possible that RBOHD-independent enzymes such as polyamine oxidase as well as cell-wall associated peroxidase (Sachdev et al. 2021; Yang et al. 2022; Wang et al. 2024) may alter apoplastic H_2_O_2_ levels and downstream signal transduction. Furthermore, Liu et al. 2022 suggested that trehalose plays a role in modulating cold stress associated with ABA signaling that is independent from H_2_O_2_. However, putative trehalose contributions to this path need further investigation. Nevertheless, the work of Han et al. (2024) represents a major advance in our understanding of trehalose and H_2_O_2_ signals in cold stress and highlights the complexity of these regulatory networks and the mechanisms involved (Fig.).

Data presented here is available in Hang et al. 2024mSID: PP2024-RA-00710R1.

## Data Availability

The data underlying this article are available in Han et al. (2024).
